# Cross-sectional study of obstetrics and gynecology-bound students in visiting rotations

**DOI:** 10.1186/s12909-025-07690-x

**Published:** 2025-07-29

**Authors:** Melody Y. Hou, Tiffany M. Hodgens, Mytien Nguyen, Marjorie J. Westervelt, Melissa A. Toland, Dowin Boatright, Claudia L. Lopez, Tonya L. Fancher

**Affiliations:** 1 Department of Obstetrics and Gynecology, University of California, Davis, 4860 Y Street, Suite 2500, Sacramento, CA 95817 USA; 2 Office of Medical Education, University of California, Davis School of Medicine, Sacramento, CA USA; 3 Yale School of Medicine, New Haven, CT USA; 4 Office of Medical Education, University of California, Davis School of Medicine, Sacramento, CA USA; 5 Evaluation, Assessment, and Medical Education Research Division, Baylor College of Medicine, Houston, TX USA; 6 Department of Obstetrics and Gynecology, University of California, Davis, Sacramento, CA USA; 7 Present address: Department of Obstetrics and Gynecology, Harbor-UCLA Medical Center, Los Angeles, CA USA; 8 Department of Emergency Medicine, New York University Grossman School of Medicine, New York, NY USA; 9 Department of Obstetrics and Gynecology, University of California, Davis, Sacramento, CA USA; 10 Office of Medical Education, University of California, Davis School of Medicine, Sacramento, CA USA

**Keywords:** Undergraduate medical education, Obstetrics and gynecology, Recruitment, Medical students, Away, Audition, Debt, Race and ethnicity

## Abstract

**Background:**

Recruitment of a more diverse obstetrics and gynecology workforce may help improve patient outcomes in the US, particularly among women of color. Visiting rotations play a role in competing for a position in an obstetrics and gynecology residency, however, not all students may be able to complete these expensive experiences. Our objective was to evaluate socioeconomic and other demographic differences among US obstetrics and gynecology-bound students who participate in visiting rotations versus those who do not.

**Methods:**

We obtained de-identified data from the Association of American Medical Colleges for students graduating in US allopathic medical schools 2019 or 2020. We analyzed self-reported receipt of state and/or federal assistance to obtain postsecondary education, medical education debt, sex, and race and ethnicity data using chi-square and ANOVA analyses and logistic regression.

**Results:**

Of 33,287 US graduating medical students, 1978 (5.9%) indicated “Obstetrics and Gynecology” as their intended practice and included socio-demographic data; 1110 (56.1%) of these completed at least one visiting rotation. In multivariable analysis controlling for medical education debt, race and ethnicity, and sex, students with moderate debt were less likely to complete any visiting rotation (aOR 0.68, 95% CI: 0.52, 0.89) and students with any debt were less likely to complete two or more visiting rotations than those without debt. However, Black students were significantly more likely to complete two or more rotations than white students when adjusted for debt and sex (aOR 1.48, 95% CI: 1.02, 2.11).

**Conclusions:**

Among US obstetrics and gynecology-bound medical students, moderate medical education debt was associated with lower odds of completing visiting rotations when adjusted for race and ethnicity and sex. Black students were more likely to complete two or more visiting rotations compared to their white counterparts when adjusted for levels of debt, perhaps to improve the likelihood of a successful match that is lower than that of their white colleagues despite the risk of worsening their debt. Providing more financial support or deemphasizing the visiting rotation as part of the application could help recruit a workforce that better reflects the diversity of the general population.

**Supplementary Information:**

The online version contains supplementary material available at 10.1186/s12909-025-07690-x.

## Background

Health outcomes improve with racial concordance between patient and physician [1, 2], but physician racial and ethnic diversity does not reflect that of the general US population [3, 4]. To improve this gap, many US healthcare systems and medical schools have focused on recruiting a more diverse workforce [5–7], yet in obstetrics and gynecology (OBGYN), the proportion of Black OBGYN residents has decreased from 2014 to 2019, with Black and Hispanic residents comprising 9.5% and 10.5% of OBGYN residents, respectively, and other specialties have not fared much better [4].

In the US, a student who completes medical school must enter a postgraduate training program, also known as a residency, to complete their training as a physician. The US residency match process involves both applicants and residency programs submitting ranked preference lists that ultimately results in the placement (or non-placement) of an applicant in a residency program [8]. Other countries employ the same process for specialty training, including the Canadian Residency Matching Service (CaRMS), United Kingdom’s Medical Specialty Recruitment, the Japan Residency Matching Program, and Abu Dhabi’s Department of Health Standard for the Allocation of Physicians in Residency Training Programs in the Emirate of Abu Dhabi (TANSEEQ) [9–12].

Many US residency program directors cite visiting rotations, also known as away or audition rotations, as one of the most influential factors for granting applicants interviews and determining their rank list in the National Resident Matching Program (NRMP) [13]. A visiting rotation is a clinical elective rotation at a different institution outside of a student’s home medical school, and can be done as individual agreements between a student and an institution, or centrally coordinated on a national level such as in the U.S or Canada [14, 15].

Visiting rotations enable students to establish a personal connection with the program. An established personal working relationship helps with a successful match. In Canada, residency selection demonstrated preference for applicants from within the same institution, and completing a visiting rotation in the U.S. and Canada potentially increases the likelihood of a student matching with the host program or within the specialty [16–18]. US medical students recognize this impact of visiting rotations [19], such that students feel obligated to complete visiting rotations to increase their chances of matching into competitive specialties, such as OBGYN [20–22]. However, 35% of US medical students are unable to participate in visiting rotations due to financial constraints, affecting low-income students and possibly racially and ethnically underrepresented in medicine (URiM) students who carry a disproportionate amount of debt [23–25]. In OBGYN, about half of residency applicants complete visiting rotations, and understanding differences between OBGYN-bound students who pursue or do not pursue visiting rotation opportunities may provide insight into the disparities seen among OBGYN residents [4, 26, 27]. Delineating these differences will be critical as the importance of a visiting rotation to match success may increase with the elimination of United States Medical Licensing Exam (USMLE) Step 1 exam numeric scores, more frequent use of pass/fail grading in required clinical clerkships, and the OBGYN specialty standards of both virtual residency interviews and application signaling [28–34]. Our aim is to identify sex, racial and ethnic, and socioeconomic factors associated with completing visiting rotations among US OBGYN-bound students. We hypothesized that OBGYN-bound students from under-represented sex, racial and ethnic, and socioeconomic backgrounds in medicine were less likely to participate in visiting rotations.

## Methods

### Study design and data source

We conducted a cross-sectional study using a de-identified dataset from the Association of American Medical Colleges (AAMC), a national membership organization representing all US allopathic medical schools. The dataset included the 2019 and 2020 Graduation Questionnaire (GQ) responses from all graduating US students [34], linked to student-level self-reported data from their American Medical College Application Service (AMCAS). The University of California, Davis Health Institutional Review Board deemed this study not human subject research.

### Setting and participants

Each year, the AAMC collects demographic data from applicants to US medical schools through AMCAS, at the time of their application. The AAMC also conducts the GQ survey of all US medical students at the end of medical school from February to June. This national survey is administered online via unique private links, with student identification remaining anonymous to schools. Respondents are not required to complete all survey items for either interface. We chose 2019 and 2020 as the final two years during which the OBGYN application process had been stable; in each year since, the OBGYN application process has been affected by responses to the coronavirus disease of 2019 (COVID-19) pandemic, US Medical Licensing Examination (USMLE) Step 1 score changes, and iterative changes prompted by the Association of Professors in Gynecology and Obstetrics’ “Transforming the UME to GME Transition: Right Resident, Right Program, Ready Day One” project [28, 30, 35].

### Variables and data measurement

Students could choose among eight race and ethnicity categories: white, American Indian/Alaska Native, Asian, Black, Hispanic, Native Hawaiian/Pacific Islander, Other, and Unknown, and could further specify with options within these categories although these were not available in the provided dataset. If students self-identified with a race(s) but did not additionally select Hispanic, we categorized them as a non-Hispanic member of that race(s). Due to small numbers in the data, we grouped students identifying as non-Hispanic American Indian/Alaska Native or non-Hispanic Native Hawaiian/Pacific Islander with Other. We assigned students who reported race and ethnicity in two or more of these groups as Multiracial. Students could disclose sex as male, female, or not disclose. Our three socioeconomic status indicators included two self-reported measures of financial assistance: receipt of Pell grant, which is a federal grant to support students obtaining postsecondary education who display exceptional financial need, and state or federal assistance, both obtained from AMCAS. Our third socioeconomic indicator was self-reported medical education debt at graduation, categorized in quintiles from zero to > $250,000, which we labeled “no debt,” “low debt”, “moderate debt”, “high debt” and “extremely high debt.” We analyzed our outcomes using responses to the following questions on the GQ: “By the time you have graduated from medical school, will you have completed any away rotations? Include only rotations that were required by your medical school for graduation AND were at institutions not affiliated with your medical school.” and “How many away rotations will you have completed? Include only rotations that were not required by your medical school for graduation AND were at institutions not affiliated with your medical school.” We excluded students who did not indicate a specialty, and who did not indicate a number for visiting rotations completed.

### Bias

We analyzed missing data from the race and ethnicity, sex, and the medical education debt questions (Appendix A) for patterns of missingness using Little’s MCAR test which yielded a chi-square statistic of 4.25 (df = 3, *p* = 0.24). Since *p* > 0.05, we considered the data missing completely at random and removed them from the sample.

### Statistical methods

We examined demographic and socioeconomic differences between students who indicated “Obstetrics and Gynecology” as their intended area of practice versus all other students in the study sample. Among students who indicated “Obstetrics and Gynecology” as their intended area of practice, we compared demographics of students who did or did not complete visiting rotations using chi-square tests and ANOVA analyses. Since visiting OBGYN-bound students complete a median of one visiting rotation [27], we evaluated those who completed 2 or more visiting rotations to identify any additional associations with completing extra visiting rotations. Reference groups were selected based on assumptions of the group whose ability to complete a visiting rotation was least likely impacted by the selected variable. We used logistic regression models to determine the association between socioeconomic status, race and ethnicity, and sex with the number of visiting rotations. We used R, version 4.3.2 for all statistical analyses [36]. A 2-sided *p* < 0.05 defined statistical significance.

## Results

The national response rate for the 2019 GQ and 2020 GQ was 83.6% and 81.6%, respectively, resulting in 33,287 responses. Students with missing data for specialty, visiting rotations, sex, medical education debt, or race and ethnicity were excluded from the analysis. Of the remaining 29,706 students, 1978/29,706 (6.6%) indicated “Obstetrics and Gynecology” as their intended area of practice. Table 1 presents the baseline demographic characteristics of the OBGYN-bound students compared to all other students in the study sample. Students intending OBGYN compared to those students who indicated a different specialty were more likely to be female, Black, Hispanic, or have medical education debt. They were also less likely to be Asian (Table 1).

**Table 1:** Demographics of and number of visiting rotations completed by students who intended to practice obstetrics and gynecology compared to all other students who completed the Association of American Medical Colleges Graduation Questionnaire in 2019 and 2020

**Variable**	**N**	**Overall**	**Ob/Gyn**	**Other Specialties**	***p*-value**^*2*^
		*N* = 29,706^*1*^	*N* = 1,978^*1*^	*N* = 27,728^*1*^	
**Sex**	29,706				< 0.001
Male		14,582 (49.1%)	244 (12.3%)	14,338 (51.7%)	
Female		15,124 (50.9%)	1,734 (87.7%)	13,390 (48.3%)	
**Race/Ethnicity**^**3**^	29,706				< 0.001
White		17,102 (57.6%)	1,151 (58.2%)	15,951 (57.5%)	
Asian		6,294 (21.2%)	297 (15.0%)	5,997 (21.6%)	
Black		1,660 (5.6%)	176 (8.9%)	1,484 (5.4%)	
Hispanic		1,598 (5.4%)	131 (6.6%)	1,467 (5.3%)	
Multiracial		2,458 (8.3%)	186 (9.4%)	2,272 (8.2%)	
Other		594 (2.0%)	37 (1.9%)	557 (2.0%)	
**Socioeconomic Status Indicators**					
Received Pell Grant^4^	27,118	5,940 (21.9%)	382 (20.5%)	5,558 (22.0%)	0.14
Received State/Fed Assistance	25,897	5,400 (20.9%)	352 (19.8%)	5,048 (20.9%)	0.26
**Medical Education Debt**^**5**^	29,706				0.006
No debt ($0)		8,775 (29.5%)	511 (25.8%)	8,264 (29.8%)	
Low ($1-$110,000)		4,037 (13.6%)	290 (14.7%)	3,747 (13.5%)	
Moderate ($110,001-$190,000)		5,789 (19.5%)	399 (20.2%)	5,390 (19.4%)	
High (190,001-$250,000)		6,299 (21.2%)	445 (22.5%)	5,854 (21.1%)	
Extremely high (> $250,000)		4,806 (16.2%)	333 (16.8%)	4,473 (16.1%)	
**Number of Visiting Rotations**	29,706				< 0.001
No Visiting Rotations		13,362 (45.0%)	868 (43.9%)	12,494 (45.1%)	
1 Visiting Rotation		6,480 (21.8%)	640 (32.4%)	5,840 (21.1%)	
2 Visiting Rotations		5,925 (19.9%)	340 (17.2%)	5,585 (20.1%)	
3 Visiting Rotations		2,941 (9.9%)	99 (5.0%)	2,842 (10.2%)	
4 + Visiting Rotations		998 (3.4%)	31 (1.6%)	967 (3.5%)	

^1^n (%)

^2^Pearson’s Chi-squared test; Wilcoxon rank sum test

^3^Due to low numbers in the cohort, students identifying as American Indian/Alaska Native or Native Hawaiian/Pacific Islander were grouped with Other race and ethnicity, and multiracial students are shown in aggregate

^4^Federal grant supporting students obtaining post-secondary education who display exceptional financial need

^5^Self-reported amount owed on medical education loans

Of the students who indicated OBGYN, 1110/1978 (56.1%) completed at least one visiting rotation (Table 1). The demographic and socioeconomic comparison of these students with those visiting students in other specialties mirrored our previous comparison of all OBGYN-bound students versus all non-OBGYN-bound students in terms of sex and race and ethnicity.

Table 2 shows that distribution of debt level varied significantly between race and ethnicity groups among OBGYN-bound students. Medical education debt was not significantly different between visiting students intending OBGYN versus other specialties (data not shown).

**Table 2:** Distribution of medical education debt by race and ethnicity of students who intended to practice obstetrics and gynecology and completed the Association of American Medical Colleges Graduation Questionnaire in 2019 or 2020

**Variable**	**White**	**Asian**	**Black**	**Hispanic**	**Multiracial**	**Other**	**Unadjusted *p*-value**^*2*^
*N* = 1,978	*N* = 1,151^*1*^	*N* = 297^*1*^	*N* = 176^*1*^	*N* = 131^*1*^	*N* = 186^*1*^	*N* = 37^*1*^	
**Med. Ed. Debt**							< 0.001
No Debt	290 (25.2%)	114 (38.4%)	19 (10.8%)	29 (22.1%)	46 (24.7%)	13 (35.1%)	
Low ($1-110,000)	149 (12.9%)	54 (18.2%)	26 (14.8%)	24 (18.3%)	32 (17.2%)	5 (13.5%)	
Moderate ($110,001-190,000)	227 (19.7%)	52 (17.5%)	39 (22.2%)	33 (25.2%)	44 (23.7%)	4 (10.8%)	
High ($190,001-250,000)	271 (23.5%)	46 (15.5%)	56 (31.8%)	29 (22.1%)	34 (18.3%)	9 (24.3%)	
Extremely High (> $250,000)	214 (18.6%)	31 (10.4%)	36 (20.5%)	16 (12.2%)	30 (16.1%)	6 (16.2%)	

^1^n (%)

^2^Pearson’s Chi-squared test

In univariate analyses of OBGYN-bound students (Table 3), we found a significant relationship between visiting rotations completed and debt level quintiles (*p* = 0.04). We found no significant difference in Pell grant or state and/or federal assistance receipt between those who completed visiting rotations and those who did not (*p* = 0.62 and 0.35, respectively). Since Pell grant and state and/or federal assistance receipt strongly correlated with medical education debt, we included medical education debt quintiles as our socioeconomic indicator for all multivariable analyses. We chose to keep sex and race and ethnicity as covariates given our aims.

**Table 3:** Demographics of students who completed a visiting rotation versus those who did not, among students who intend to practice obstetrics and gynecology and completed the Association of American Medical Colleges Graduation Questionnaire in 2019 or 2020

**Variable**	**N**	**No Visiting Rotations**	**Any (1 +) Visiting Rotations**	**Unadjusted *p*-value**^*2*^
		*N* = 868^*1*^	*N* = 1,110^*1*^	
**Sex**	1,978			0.06
Male		121 (13.9%)	123 (11.1%)	
Female		747 (86.1%)	987 (88.9%)	
**Race/Ethnicity**^**3**^	1,978			0.59
White		521 (60.0%)	630 (56.8%)	
Asian		118 (13.6%)	179 (16.1%)	
Black		76 (8.8%)	100 (9.0%)	
Hispanic		53 (6.1%)	78 (7.0%)	
Multiracial		83 (9.6%)	103 (9.3%)	
Other		17 (2.0%)	20 (1.8%)	
**Socioeconomic Status Indicators**				
Received Pell Grant^4^	1,860	171 (21.0%)	211 (20.2%)	0.68
Received State/Fed Assistance	1,780	160 (20.6%)	192 (19.2%)	0.50
**Medical Education Debt**^**5**^	1,978			0.04
No debt ($0)		204 (23.5%)	307 (27.7%)	
Low ($1–110,000)		133 (15.3%)	157 (14.1%)	
Moderate ($110,001–190,000)		198 (22.8%)	201 (18.1%)	
High ($190,001–250,000)		196 (22.6%)	249 (22.4%)	
Extremely high (> $250,000)		137 (15.8%)	196 (17.7%)	

^1^n (%)

^2^Pearson’s Chi-squared test

^3^Due to low numbers in the cohort, students identifying as American Indian/Alaska Native or Native Hawaiian/Pacific Islander were grouped with Other race and ethnicity, and multiracial students are shown in aggregate

^4^Federal grant to support students obtaining post-secondary education who display exceptional financial need

^5^Self-reported amount owed on medical education loans

**Fig. 1:**
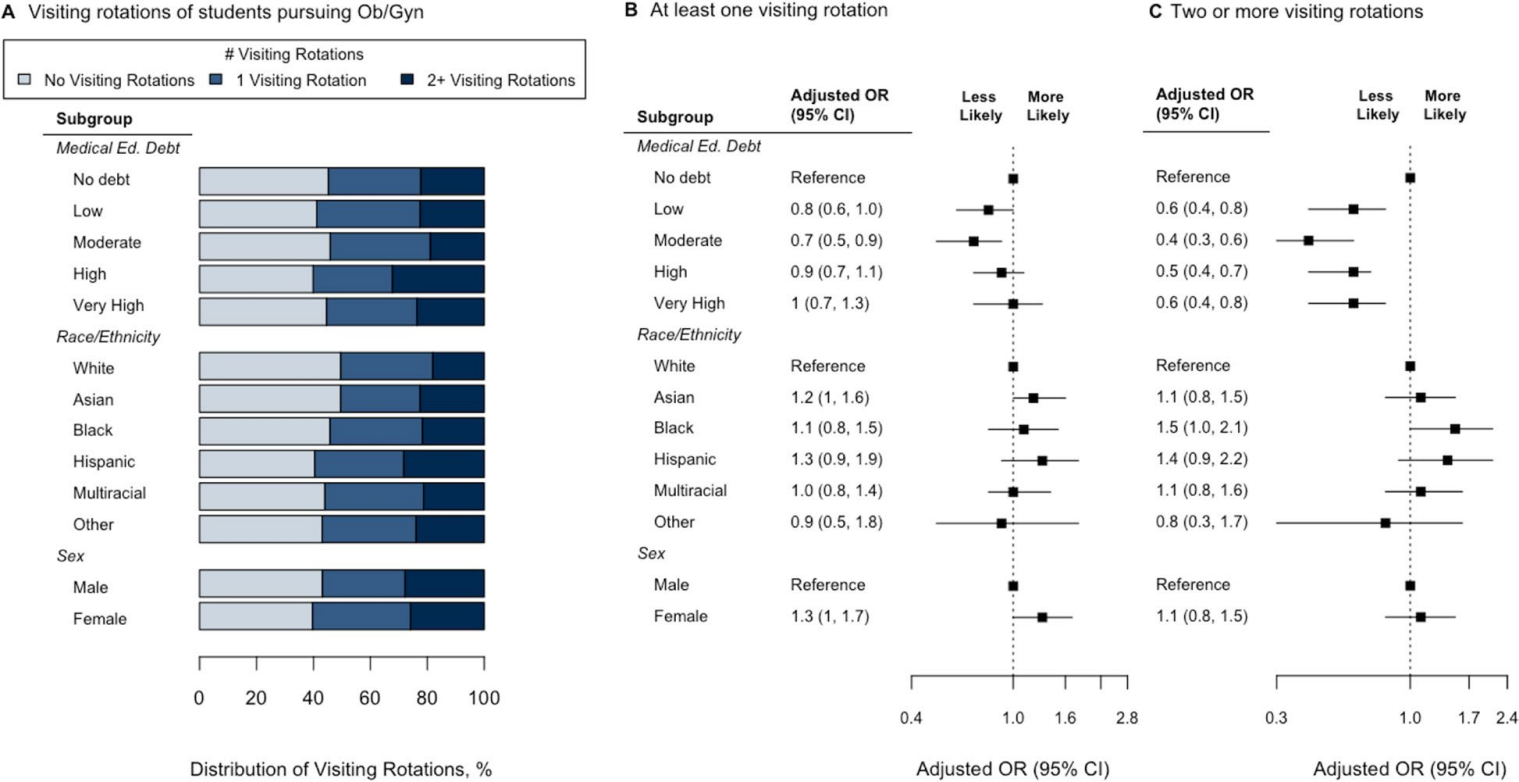
Associations with the number of visiting rotations completed among medical students who completed the Association of American Medical Colleges Graduation Questionnaire in 2019 or 2020 who intended to practice obstetrics and gynecology. (A) Distribution of visiting rotations (light blue = none, medium blue = 1, dark blue = 2 or more) by medical education debt quintile, race and ethnicity, and sex; medical education debt quintile definitions: no debt, $0; low, $1–110,000; moderate, $110,001–190,000; high, $190,001–250,000; extremely high, > $250,000 (B) Adjusted odds ratios of completing any visiting rotation or (C) two or more visiting rotations, by medical education debt quintile, race and ethnicity, and sex, using white males with no medical education debt as the reference group. Due to low numbers in the cohort, students identifying as American Indian/Alaska Native or Native Hawaiian/Pacific Islander were grouped with Other race and ethnicity, and multiracial students are shown in aggregate. Medical Ed Debt, medical education debt is self-reported amount owed on medical education loans

In multivariable logistic regressions using medical education debt quintiles, race and ethnicity, and sex, having moderate debt was associated with lower odds of completing any visiting rotation (aOR 0.68, 95% CI: 0.52, 0.89; *p* = 0.01), and any (non-zero) medical education debt was associated with lower odds of completing two or more visiting rotations (all with *p* < 0.002) (Fig. 1). However, when adjusted for debt and sex, Black students were significantly more likely to complete two or more visiting rotations than white students (aOR 1.48, 95% CI: 1.02, 2.11; *p* = 0.03).

## Discussion

Visiting rotations are considered to have significant influence in the competitive process of matching into a US residency [13]. In 2023 dollars, the mean estimated cost for a single visiting rotation is $1240 exclusive of the cost of maintaining a primary residence [37, 38]. More than half (56%) of US OBGYN residency applicants in our study completed visiting rotations, of which 42.3% completed two or more. We found that OBGYN-bound medical students with moderate levels of medical education debt ($110,001 to 190,000) were less likely to complete one or more visiting rotations. When adjusted for race and ethnicity and sex, students with non-zero medical education debt were less likely to complete two or more visiting rotations. When we adjusted for medical education debt and sex, Black students were significantly more likely to complete two or more rotations than white students.

Researchers have shown that an unsuccessful match can be related to racial, sex and gender disparities in both the US and Canadian match process [26, 39–41]. In Japan, poorer exam scores have been associated with an unsuccessful match, but exams themselves may be compromised by implicit biases [42, 43]. Although one Canadian study evaluated the relationship between visiting rotations and match success in otolaryngology [18], research investigating this relationship in non-US countries or investigating the factors associated with completing visiting rotations has been limited. Countries with centralized coordination of visiting rotations, such as Canada with its AFMC Student Portal, coordinated by the Association of Faculties of Medicine of Canada [15], should evaluate their national data for evidence of population-level disparities, and institutions who independently offer visiting rotations should examine their institutional data as well.

Although we demonstrated a clear association between any level of medical education debt and not completing two or more visiting rotations, the relationship between debt and odds of completing any visiting rotation was not linear and will require additional research. In our study, Black students were more likely to complete two or more visiting rotations compared to their white counterparts when adjusted for levels of debt, which suggests that Black students with equal educational debt seek out more visiting rotations than their white colleagues. More study is needed on why Black students are more willing to bear the cost and time burden of visiting rotations. This may be driven by students with limited professional networks [44] who complete visiting rotations for a better opportunity to assess potential residency programs rather than relying on programs’ online self-portrayal [19]. However, this difference may instead reflect students completing visiting rotations in an attempt to increase their chance of successful match [13]. Other research demonstrates that URiM students have lower rates of transition into graduate medical education [26], and that these students already bear a disproportionate amount of debt [24, 25]. URiM students with debt may exacerbate their debt by completing more visiting rotations which ultimately may not improve their match success: despite visiting rotations being influential, only 34.7% of OBGYN program directors guarantee an interview to students on a visiting rotation and less than 25% of residents in most OBGYN residencies completed a visiting student rotation with their program [45]. We are also concerned that URiM students with debt who are advised to complete more visiting rotations to improve their chances of matching in OBGYN may decide not to apply to the specialty due to the considerable financial investment required [26, 46]. These hypotheses require additional investigation.

Residency programs can take several actions in response to these findings. Programs should offer more clarity regarding the impact of visiting rotations on an applicant’s interview invitation and matching chances, as the Coalition for Physician Accountability has called for transparency from residency programs about how visiting rotations are used for interview and resident selection [45, 47]. Although the OBGYN specialty has committed to virtual residency interviews to help mitigate financial inequities among students [30], no such commitment has been made regarding visiting rotations. The scant existing scholarships offered to low-income students to complete rotations may become even more limited in the current political climate in the US [48]. Expanding the number of schools offering need-based debt-free medical education or short term zero-interest loans may remain aspirational goals [49].

Individual programs instead can strengthen their mission-based holistic reviews of applications, which is a foundational strategy to promoting diversity, equity, and inclusion in residency [50], and deemphasize the weight of the visiting rotation in the application process. The OBGYN specialty society can create residency program partnerships with Historically Black Colleges and Universities (HBCUs) and other Minority Serving Institutions to improve networking opportunities for those students [44, 51, 52] and consider instituting a cap on the number of visiting rotations a student can complete. Ensuring equitable opportunities to all students throughout medical school [50], including visiting rotations, is critical for recruitment of diverse students.

Our study has several limitations. Our cross-sectional study is only able to establish associations with visiting rotation completion but not causality, and these self-reported data are subject to response bias. We excluded students with missing data, although data appeared to be missing at random, and we were not powered to draw conclusions for the “other” group in race and ethnicity analyses. We also lack student performance metrics, such as grades and USMLE Step scores, geographic location, and evidence of financial or cultural institutional support, which may influence the decision to participate in visiting rotations. Finally, these data are not linked to students’ actual match outcomes, data which are owned by the NRMP, an independent organization not associated with AAMC. Our findings with 2 years of data are insufficient to establish a trend, and may not be generalizable to current practices, since the visiting rotation moratorium in academic year 2020–21 due to COVID-19, [35] the limit of one visiting rotation per student in the following academic year 2021–22, USMLE Step 1 now graded as pass or fail [28], more medical schools moving to pass/fail clerkship grading [29], and virtual residency interviews [30] may have other effects on the perceived value of visiting rotations. Since the latter three issues may lead to challenges with applicant evaluation and differentiation for residency programs [32, 33, 53], program directors may place even more weight on visiting rotations. Our study thus plays a critical role by identifying disparities in medical education debt and race and ethnicity in visiting rotation completion, which may be exacerbated if program directors increase the emphasis on visiting rotations.

## Conclusions

Among obstetrics and gynecology-bound medical students, moderate medical education debt was associated with lower odds of completing visiting rotations pre-COVID-19, with non-zero debt associated with lower odds of completing 2 or more visiting rotations compared with students with no debt. However, Black students were more likely to complete two or more visiting rotations than white students, adjusted for debt and sex. We are concerned that this difference may reflect the willingness of these students, who are at risk of bearing a disproportionate amount of debt, to exacerbate their debt by completing more visiting rotations to improve their disproportionate lack of success in matching. The post-pandemic changes to the OBGYN application process, including Step 1 and clerkship grade changes and virtual interviews, may increase the importance of visiting rotations and further exacerbate these disparities despite our efforts to diversify our workforce. As matching into the specialty becomes more competitive, either providing more financial support or conscientiously deemphasizing or capping the visiting rotation as part of the application will be critical in our goal for parity.

## Supplementary Information

Supplementary Material 1.

## Data Availability

The data that support the findings of this study are available from the Association of American Medical Colleges (AAMC) but restrictions apply to the availability of these data, which were used under license for the current study, and so are not publicly available.

## Abbreviations

OBGYN: Obstetrics and gynecology
NRMP: National Resident Matching Program
URiM: Underrepresented in medicine
AAMC: Association of American Medical Colleges
GQ: Graduation Questionnaire
AMCAS: American Medical College Application Service
Coronavirus: Disease of 2019, COVD-19
USMLE: US Medical Licensing Exam
