# Supplementary material for: Cross-sectional study of obstetrics and gynecology-bound students in visiting rotations
Source: BMC Med Educ. 2025 Jul 29;25:1125. doi: 10.1186/s12909-025-07690-x (PMC12305918; doi:10.1186/s12909-025-07690-x)
Supplement: Supplementary file 1 — Supplementary Material 1. [file 12909_2025_7690_MOESM1_ESM.docx]

Appendix A

Selected questions extracted from Association of American Medical Colleges (AAMC) Graduation Questionnaire (GQ), 2019-2020:

- When thinking about your career, what is your intended area of practice?
- By the time you have graduated from medical school, will you have completed any away rotations? Include only rotations that were not required by your medical school for graduation AND were at institutions not affiliated with your medical school.
- How many away rotations will you have completed? Include only rotations that were not required by your medical school for graduation AND were at institutions not affiliated with your medical school.
- Do you have any outstanding education loans (including loan service commitments) for your medical school education?
- Enter the amount you owe on your medical education loans. Principal amount borrowed (do not include interest):
